# Prevalence of Proton Pump Inhibitor Use Among Patients With Cancer

**DOI:** 10.1001/jamanetworkopen.2021.13739

**Published:** 2021-06-16

**Authors:** Jean-Luc Raoul, Catherine Guérin-Charbonnel, Julien Edeline, Victor Simmet, Marine Gilabert, Jean-Sébastien Frenel

**Affiliations:** 1 Department of Medical Oncology, Institut de Cancérologie de l’Ouest, Saint-Herblain, France; 2 Oncology Data Factory & Analytics, Institut de Cancérologie de l’Ouest, Saint-Herblain, France; 3 Department of Medical Oncology, Institut Centre E. Maquis, Rennes, France; 4 Department of Medical Oncology, Institut de Cancérologie de l’Ouest, Angers, France; 5 Department of Medical Oncology, Institut Paoli-Calmettes, Marseille, France

## Abstract

This cross-sectional study evaluates the prevalence of proton pump inhibitor use among patients with cancer at 4 French comprehensive cancer centers.

## Introduction

The cancer armamentarium is evolving with evidence of the efficacy of oral treatments. Their mode of administration is simple and convenient, but absorption is influenced by diet and gastric pH. Moreover, the efficacy of checkpoint inhibitors (CPIs) depends on the gut microbiome. Proton pump inhibitors (PPIs), which are among the most widely prescribed drugs, decrease the bioavailability of oral cancer treatments, lowering their efficacy,^1,2,3,4^ and induce major microbial alterations in the gut.^4^ We conducted a study to evaluate the prevalence of PPI prescribing, to identify the factors associated with PPI prescription, and to focus on patients receiving tyrosine kinase inhibitors (TKIs), CPIs, and capecitabine.

## Methods

This cross-sectional study was conducted in 4 French Comprehensive Cancer Centers from June 15 to 19 and from June 22 to 26, 2020. It was approved by the ethics committee of Angers University, which waived the need for informed consent in accordance with university policy. This study follows the Strengthening the Reporting of Observational Studies in Epidemiology (STROBE) reporting guideline.

Volunteer physicians collected data from patients with cancer seen in consultation or in the Same Day Unit. An information sheet (explaining the goals of the study, that patients could refuse to participate, and that all data would be anonymized) was given to patients; those who accepted were included. The physicians then completed a form (with scrolling menu) based on the patient’s medical records and answers to a few questions.

Parameters were compared using Welch *t* test, χ^2^ test, or Fisher exact test as appropriate. Significant parameters were entered into a multivariable logistic regression with a stepwise procedure. All *P* values were 2-sided with *P* < .05 considered significant. Statistical analyses were performed with R statistical software version 4.0.2 (R Project for Statistical Computing).

## Results

In total, 872 complete and verified (data manager) forms corresponding to 566 women (64.9%) and 306 men (35.1%) (median [range] age, 63 [20-91] years) (Table 1) were recorded. Among these, 229 patients (26.3%) were taking PPIs on the day of inclusion. Most patients who used PPIs did so on a regular basis (163 patients [71.1%]), at normal dosage (154 patients [67.2%]), for epigastric pain (114 patients [50.0%]), retrosternal pain (32 patients [14.0%]), proven esophageal or gastric ulcer (18 patients [8.0%]), or gastroprotection (34 patients [15.0%]).

**Table 1.** Demographic and Clinical Characteristics of Patients With Cancer by PPI Use

Characteristic	Total patients, No.	Patients, No. (%)		*P* value	Multiparametric analysis, OR (95% CI)
		Do not use PPIs^a^	Use PPIs		
Total patients	872	643 (73.7)	229 (26.3)	NA	NA
Age, median (range), y	63 (20-91)	62 (20-91)	66 (20-90)	<.001	1.02 (1.00-1.03)
Sex, No. (%)					
Female	566 (64.9)	425 (75.1)	141 (24.9)	.25	NA
Male	306 (35.1)	218 (71.2)	88 (28.8)		
Center					
Angers	64	43 (67.2)	21 (32.8)	<.001^b^	2.10 (1.04-4.23)
Marseille	55	29 (52.7)	26 (47.3)		3.49 (1.71-7.22)
Nantes	590	437 (74.0)	153 (26.0)		1.51 (0.94-2.53)
Rennes	163	134 (82.2)	29 (17.8)		1 [Reference]
Eastern Cooperative Oncology Group performance status					
0	407	332 (81.6)	75 (18.4)	<.001^b^	1 [Reference]
1	367	248 (67.6)	119 (32.4)		1.92 (1.36-2.72)
2-3	75	45 (60.0)	30 (40.0)		2.51 (1.41-4.45)
Unknown	23	18 (78.3)	5 (21.7)		NA
Tumor extension					
Localized	295	234 (79.3)	61 (20.7)	.03^b^	NA
Locally advanced	103	75 (72.8)	28 (27.2)		
Metastatic	474	334 (70.5)	140 (29.5)		
Primary tumor					
Breast	349	271 (77.7)	78 (22.3)	.045^b^	NA
Gastrointestinal	150	102 (68.0)	48 (32.0)		
Urologic	96	66 (69.0)	30 (31.0)		
Thorax	69	45 (65.2)	24 (34.8)		
Gynecologic	58	40 (69.0)	18 (31.0)		
Brain	47	36 (77.0)	11 (23.0)		
Hormonotherapy	125	101 (80.8)	24 (19.2)	.01	0.59 (0.37-0.90)

Abbreviations: NA, not applicable; PPI, proton pump inhibitor.

^a^ Percentages are calculated across the row, not down the column.

^b^ The distributions between PPI users and nonusers were significantly different.

In univariable and multivariable analyses, the factors associated with PPI use were age (odds ratio [OR], 1.02 [95% CI, 1.00-1.03]; *P* < .001), center (reference, Rennes; Angers: OR, 2.10 [95% CI, 1.04-4.23]; Nantes: OR, 1.51 [95% CI, 0.94-2.53]; Marseille: OR, 3.49 [95% CI, 1.71-7.22]; *P* < .001), Eastern Cooperative Oncology Group performance status (PS) (reference, PS 0; PS 1: OR, 1.92 [95% CI, 1.36-2.72]; PS 2: OR, 2.51 [95% CI, 1.41-4.45]; PS 3: OR, 2.33 [95% CI, 0.32-12.33]; *P* < .001), hormone treatment (OR, 0.59; 95% CI, 0.37-0.90; *P* = .01), metastatic stage (χ^2^_2_ = 7.42; *P* = .03), and tumor site (*P* = .045, Fisher exact test). The prevalence of concomitant PPI use differed with the cancer treatments (Table 2). Among the 20 patients taking TKIs and PPIs, 16 reported the long-term use of PPIs, mainly for epigastric pain (11 patients). If we focus on the 134 patients receiving drugs known to decline in efficacy when given with PPIs, 39 (29%) had concomitant prescriptions: capecitabine (5 of 21 patients), sunitinib (5 of 9 patients), cabozantinib (2 of 3 patients), pazopanib (1 of 5 patients), gefitinib (1 of 1 patient), erlotinib (1 of 2 patients), and sorafenib (1 of 3 patients), as well as 23 of 90 patients (25.6%) receiving CPIs.

**Table 2.** Prevalence of PPI Use per Treatment Most Frequently Received

Treatment	Total patients, No.	PPI users, No. (%)
Intravenous chemotherapy	475	130 (27.4)
Checkpoint inhibitors	90^a^	23 (25.6)
Tyrosine kinase inhibitors	60	20 (33.3)
Hormones	125	24 (19.2)
Oral chemotherapy	42	8 (19.0)
Capecitabine	21	5 (23.8)

Abbreviation: PPI, proton pump inhibitor.

^a^ Eleven patients had concomitant checkpoint inhibitor use and intravenous chemotherapy, and 3 patients using both tyrosine kinase inhibitors and checkpoint inhibitors were counted in both groups.

## Discussion

In this cross-sectional study, more than one-quarter of the patients (predominantly older with poorer PS) used PPIs. Almost one-third of those treated with drugs suspected to have altered efficacy if taken concomitantly with PPIs received PPIs.

This study has some limitations. Not all physicians agreed to participate and did not include all patients seen (data collection was time-consuming). However, the study was prospective, with no major bias, and included many patients (872 patients). The high frequency of PPI use in Marseille may be associated with the fact that the participating physicians were largely treating pancreatic adenocarcinomas.

The long-term use of PPIs is a problem in the population of patients with cancer, who may have various comorbidities, such as kidney dysfunction, anemia, infection, or hypomagnesemia.^5^ Moreover, indirect drug-drug interactions (eg, lower absorption of TKIs and capecitabine and gut microbiome changes) are associated with a decrease in survival benefit for some of these drugs.^1,2,3,4^ PPIs should be actively identified and substituted. In the case of heartburn or epigastric pain, antacids should be used; H2 antagonists are another option. TKIs must be taken 2 hours before or 10 hours after H2 antagonists. If PPI use is mandatory, TKIs must be given in the morning 2 hours before PPIs, or with an acidic beverage (eg, cola). For patients taking CPIs, the use of antacids is the best option.
